# Investigating the “Embodied Spaces of Health” in Marathon Running: The Roles of Embodiment, Wearable Technology, and Affective Atmospheres

**DOI:** 10.3390/ijerph19010043

**Published:** 2021-12-21

**Authors:** Yi Ouyang, Xiaomei Cai, Jie Li, Quan Gao

**Affiliations:** 1 School of Geographical Sciences, Guangzhou University, Guangzhou 510006, China; ouyangyi@gzhu.edu.cn (Y.O.); jieli@gzhu.edu.cn (J.L.); 2 School of Tourism Management, South China Normal University, Guangzhou 510006, China; caixm@scnu.edu.cn; 3 School of Geography and Planning, Sun Yat-Sen University, Guangzhou 510006, China

**Keywords:** running, bodily space, affective atmospheres, health

## Abstract

This paper examines how spaces of health are produced through embodied and affective practices in marathon running in China. While the social-cultural effects of distance running have gained increasing attention among public health scholars and policymakers, there has been little effort paid to the spatiality of running and its contributions to producing healthy spaces for the general public. This paper therefore fills the lacuna through a qualitative study that was conducted with 29 amateur marathon runners in China. Drawing on the Gioia Methodology in coding and analyzing qualitative data, we highlight the interactive effects of body, wearable technology, and affective atmospheres in producing what we call “embodied space of health.” We suggest that the embodied space of health is not simply the bodily experience per se but rather a relational space constituted through the co-production of body, non-human objects, and space/place. It is through these relational spaces that the effects of health and well-being (e.g., self-exploration and therapeutic feelings) emerge in marathon.

## 1. Introduction

Running has gradually become an important inquiry to public health researchers and policymakers since the late 1970s given its health-promoted benefits [1,2]. In particular, over the past one decade or so, scholars have shown growing interests in exploring the cultures of distance running and how they relate to the issues of health and wellbeing [3,4,5,6,7]. This cultural approach to running studies pay attention to the ways in which healthy lifestyles are produced and maintained through running cultures and practices [6,8]. They contend that running is a form of embodied practice through which people’s health consciousness and subjectivity are shaped. For example, distance running is increasingly perceived by people as a way to attain self-realisation and a self-disciplined lifestyle [4]. However, existing research in the cultural studies of running largely neglects the spatial dimension of running and especially how the consequence of health emerges from the interaction between body, object, and space/place in running. Some scholars have noted that space/place matters in health studies not only because some places (e.g., therapeutic landscape) have health-promoted effects but because space conditions and mediates people’s practices of health making [9,10]. This is particularly the case in running, an inherently spatial practice that calls upon the body to move across/through spaces [4]. Nevertheless, the relationship between running, space, and health warrants a closer examination.

This paper therefore fills the lacuna through elaborating on the idea of what we call “embodied space of health” and through a qualitative study of marathon running in China. Over the past decade, marathon running has become one of the most popular sports in China. The number of marathon races held on the country has dramatically grown from 22 in 2011 to 1581 in 2018 [11]. During the same period, the number of participants has also increased from 410 thousand to more than 7 million [11]. Marathons have gained much attention from not only the public but also the policymakers. In 2017, the Chinese state launched a Marathon Development Plan, which signified that the marathon was promoted to a state-sanctioned program [11]. According to Ronkainen et al., the growing demands and enthusiasm for marathon running in China is fueled by the country’s socio-political reform and the dramatic economic growth over the past two decades [12]. The significant improvement on people’s life quality has led to “a rapidly growing, health-conscious, and affluent class” who have paid increasing attentions to their bodies and health [12] (p. 42). In the ideological sphere, the market-oriented and neoliberal reforms in China have released people from the constraints of collectivist ideologies, which instead enables individuals’ pursuit of self-enrichment and self-making [13]. In particular, health and health-making are viewed as an important facet of desired citizenship and particularly individuals’ utility in a market-oriented society [13]. The popularity of marathon in China has opened up new spaces for health practices. These social contexts provide an important entry to examine the social-cultural logics of marathon and especially the interaction of body, space, and health in the marathon. Against this backdrop, this paper therefore aims to explore how spaces of health are produced through the embodied and affective practices in running. It is noteworthy that we utilise “space” rather “place” in this paper because space addresses a more relational account of health-making, while place is often associated with particular qualities of health (e.g., therapeutic landscape). Nevertheless, we suggest that space can become place through embodied and affective practices in running. Drawing on Gioia Methodology [14], we emphasise the interactive effects of body, wearable technology, and affective atmospheres in producing the “embodied space of health.” This paper therefore moves away from a biophysical study of health in running studies to an embodied and relational account of running and health.

## 2. Recreational Running and the Embodied Space of Health

### 2.1. Recreational Running, Space, and Health

Public health scholars in recent years have shown growing interest in exploring the role of recreational running in shaping the practices and subjectivities of health [4]. Running is perhaps one of the most popular form of sports that contributes to a healthy lifestyle [2]. In a medical sense, running positively facilitates the achieving of healthy body through tackling obesity, improving well-being and mental health status, inspiring individuals’ desires for participating in sports activities, and increasing people’s capacity of self-management [15]. The popularity of running is not only due to its health-related benefits but also the low costs of entry that enable individuals to easily and inexpensively practice at flexible space-times [16,17]. Moreover, running as a widely participated sport is increasingly promoted by the state to build up a “healthy society,” which is integral to the neoliberal health policy that channels the accountability for health from state to individuals [18]. Therefore, running is an important topic for public health policymakers around the world.

In particular, the cultural studies of recreational running has gained growing attentions in recent years [3,6,7,19]. This line of research primarily focuses on how health practices and subjectivities are constituted through social construction of running. For example, Collinson and Hockey suggested that distance running can create subculture and collective identity within runners in which particular values and dispositions, such as self-discipline, stoicism, and sportsmanship, are valorized [3]. As they note, the “affective community of friends and fellow runners” influences runners’ social-psychological capacity to manage their bodies and especially injuries and restoration [3] (p. 394). Yet, the sociality of running is not only shaped by the collective practices and interactions of runners but also the spatial-temporal arrangements and the collective physical infrastructures that structures runners’ health practices [20,21]. In general, running is argued to be a key element of the self-realisation and identity-making of its practitioners who seek to demonstrate their skills and affirm their beliefs in healthy lifestyle: “this identity extends beyond immediate benefits, such as body tone, weight loss, and overall fitness, and comes to include a more intrinsic self-identification as a runner” [7] (p. 338). Another strand of research relates running to the production of healthism and health-related consciousness [6,14]. Runners’ health practices and desires for fitness is also influenced by the discourses of healthism constructed by society and media on the “internalized body-ideals—on what the healthy and fit body should look like, and how to gain body-satisfaction” [6] (p. 18). The promotion of healthism in many societies has become a device to achieve social control and to produce productive, healthy, and self-responsible citizens. In this sense, social and political contexts are also crucial in shaping the health practices and subjectivities of runners, as running is a site through which healthism is internalised into runners’ body.

Although existing scholarship has acknowledged the cultural significance of running, little attention has been paid to the spatiality of running and especially how running can create health-promoting spaces [4]. In essence, running is an inherently spatial practice because it calls upon the body to move across/through space, place, and landscape and influences the way individuals make sense of the environments around them [18]. Some research has revealed that space/place matters in eliciting running practices and public health [22,23,24]. This primarily manifests in the studies that examine the significance of events (e.g., road runs and fun runs) and particular place/landscape (e.g., parks, forests, and hill) in stimulating the health effects of running [22,23,24]. Nevertheless, a more nuanced understanding of running, space, and health remains piecemeal [21]. Attending to the spatiality of running and health should focus on not only the body as a site through which health subjectivities are formed but also how the embodied practices of running may create new spaces of health [4,21]. In other words, embodiment is not only a crucial dimension of health but also influences the ways that runners make sense of the space and place around them [4]. This paper therefore contributes to this inquiry by elaborating on what we call the “embodied spaces of health” in recreational running.

### 2.2. Theorising the “Embodied Space of Health” in Running

It has been widely recognised that running is an embodied and mobile practice that moves in/across space/place [4]; yet, how health subjects form and emerge from the interaction between body and space is largely under-theorised. Some research has revealed that health and well-being are not simply acquired through the management of medical body but also cultivated through the encounters with particular space/place, for instance, the therapeutic landscapes [10]. The importance of the concept of space/place in health studies is further acknowledged by the relational approach to health studies that draws upon the merits of actor-network theory [25]. This approach argues that effects of health “emerge from relationalities, interactions and assemblages of body/self, social discourses, more-than-human subjects, and the broader social-environmental setting” [26] (p. 1). Bearing this in mind, running is better understood as an embodied encounter between people and space/place [27]. In running, people and space are actually in “a mutually reinforcing and reciprocal relationship” [27] (p. 1825). For example, Little’s study of running in nature suggested that people’s intimacy with nature is integral to their project of self-caring, while the running practices in turn produce an authentic natural space that is emotionally perceived as health-promoted [4]. This paper therefore advances the relational and spatial approach to running and health by introducing the concept of what we call “embodied space of health.” There are at least three lines of insights that contribute to our theorisation of the “embodied space of health.”

First, the “embodied space of health” recognises the role of bodies and bodily practices at the centre of health making and the production of health-promoted space/place. The health effects of running are not instinctive but rather learned and disciplined through running bodies such that particular health subjectivities, consciousness, dispositions, and lifestyles can become part of the self [4,28]. Hanold pointed out that the bodily experiences of pain during marathon running provide a way for the runners to explore the capacity of the body and to achieve self-realisation [29]. He also suggested that the desires for healthy and disciplined bodies reproduce the social norms that associate marathon running with middle-classness. Similarly, inspired by Lefebvre’s rhythm analysis, Edensor and Larsen examined the bodily rhythms of marathon practitioners [5]. They argued that marathon running is a rhythm that is collectively achieved by the spatial-temporal arrangements of the body, place, environment, and various actors. Therefore, runners need to train and manage their body in order to mobile to attain mobile rhythms and “experience a collective eurhythmia with fellow runners” [5] (p. 731). In short, marathon running involves management of the desires, capacity, pain, and rhythm of the body through which the health subjects can emerge.

Second, existing literature of running has begun to acknowledge the role of wearable technologies (e.g., smartwatch, self-tracking devices) in the production of health-promoting places/spaces [4,30,31]. The literature on the “quantified self” has suggested that digital technologies can help quantify bodies and their interaction with places to facilitate self-betterment and self-reflection [32]. This is particularly the case in the wide utilisation of wearable technologies, such as self-tracking devices in jogging and running. Esmonde’s study of women’s use of fitness tracking technologies indicated that digital technologies can enhance, reframe, or even undermine the pleasure that runners derive from their body’s movement through space. She further acknowledges the non-human agency of digital technologies that data collection in turn disciplines individuals’ feelings of health [31]. Little [4], however, argued that some runners’ use of digital technology may influence personal values of health that cannot be quantified, such as sociality and escape from regular life patterns. Nevertheless, human, non-human, and other types of objects are capable of acting and shaping the social-spatial relations of health. Wearable technologies therefore can be considered as an important factor in shaping the “embodied space of health” in running.

Third, the “embodied space of health” captures not only the bodily experiences per se but also the diffused, ambiguous, and non-representational spaces of health. This is particularly the case in the studies of “atmospheres,” “affect,” and “moods” of running [33,34]. Atmosphere is often understood as the “spatially extended quality of feeling” and “something distributed yet palpable, a quality of environmental immersion that registers in and through sensing bodies whilst also remaining diffuse, in the air, ethereal” [35] (p. 413). Researchers often use the concept of “affective atmospheres” to understand this diffused forms of spatiality. “Affective atmospheres” are not feelings and bodily experiences per se but something formed through the interactions between bodies, objects, and environments, which in turn have the capacity to shape and condition people’s behaviours and subjectivities [36,37]. Lupton contended that affective atmospheres can profoundly influence the ways in which people “sense the spaces they inhabit and through which they move and the other actors in those spaces;” therefore, affective atmospheres also shape how health is felt and performed in specific spaces [32] (p. 1). For example, Larsen and Jensen considered weather as an important affective atmosphere in distance running [37]. They contended that “concrete and situated weather conditions are felt in our multi-sensorial embodied relations to the ‘outer environment’” so that the experiences of running bodies can be animated [37].

In general, existing literature has indicated that body, technology, and atmospheres are important in shaping the space of health. However, there is scant research that examine how body, technology, and atmospheres mutually shape one another in a way that may engender new subjectivities and spaces of health in running. This paper therefore aims to explore how embodied spaces of health are produced through the interaction of body, non-human actors, and environments.

## 3. Materials and Methods

### 3.1. Data Collection

The materials of this paper are based on a project that explores the embodied practices of marathon runners in China. Our data were collected through a qualitative study conducted from September 2019 to January 2020. The methods utilised in this study included participant observation and in-depth interviews. We also collected four runners’ dairies that recorded their experience during marathons. The dairies were copied, with respondents’ permission, and analysed as important data sources of this research project. The interviews were conducted with 29 amateur marathon runners, including 20 men and 9 women, with ages varying from 22 to 55 (see Table 1). All respondents had participated in at least two marathons in one year. Most respondents were well-educated university students, managers, or professionals who can be roughly categorised as middle class in China. The interviews were largely unstructured to encourage participants to freely narrate their experiences of running and how they make sense of the environments around them during running. Interviews lasted from 30 min to 2 h and were recorded and transcribed in full. Pseudonyms are utilised to protect the anonymity of respondents.

### 3.2. Data Coding and Analysis

This paper engages the Gioia Methodology to analyse and interpret interview and dairies data so as to increase the “qualitative rigor” in inductive research [14]. This methodology is a modified version of grounded theory that aims to reveal the structure and connection of qualitative data through conceptualisation and coding. There are some basic steps of this method. First, researcher should “start looking for similarities and differences among emerging categories” and “bend over backward to give those categories labels that retain informants’ terms” [38] (p. 286). Second, we consider the constellation of 1st-order codes, which should adhere faithfully to the terms ustilised by the informants. Third, if there are some deeper process or structures underlying the 1st-order codes, we then can proceed to 2nd-order themes and aggregate dimensions that form the basis for building a data structure [14]. The interview and dairies data were coded with the assistance of the qualitative data analysis software NVivo 11. As a result, we generated 522 codes that relate to “body, space, and health” in marathon running. Based on these 522 codes, we conducted 1st-order analysis, which resulted in eleven 1st-order concepts and six 2nd-order themes. An overview of the coding is presented below in Table 2.

## 4. Results

### 4.1. Marathon Running and the Bodily Experience of Health

The perception and exploration of the body is central to the health effects of marathon running. Our coding processes show that the bodily experience of health can be divided into two conceptulisations of the body: First, runners attempt to build up the capacity of the body by cultivating healthy body and exploring the potentials of their body; second, through marathon running, practitioners reclaim the autonomy of the body that was thwarted by the programmatic lifestyles and social norms in the city. In general, the body is a crucial site through which not only the biophysical presence of health but also the health subjectivities are formed.

Pursuing a healthy body or desired body shape is often one of the main motivations for participating into marathon running. However, marathon is nevertheless an intensive endurance sport that may not be suitable for those who are not ready for a full marathon. Therefore, many practitioners would engage in normal running first as pre-marathon training and consider participating into marathon as the impetus to push them to build up a healthy lifestyle and disciplined body. For example, M4, a 40-year-old teacher who was troubled by obesity, told us:

You know, medically speaking, running is the best cure for illness. I have hyperlipidaemia and fatty liver. So, I decided to change by running marathon. But I am not ready for a full marathon. I just ran around in the playground and hopefully I will be ready to participate one day. Despite this, I can see significant change that took place on myself. I became more self-disciplined. To prepare for the marathon, I have a morning jog and regular diet every day.

When we re-interviewed M4 four months later, he had completed his first marathon attempt. However, we suggest that people’s engagements with marathon often goes beyond concerns for the biophysical sense of health but also contributes to the construction of a running body through which to achieve self-exploration. Many runners highlight that marathon is a journey of self-exploration and transformation in which you can truly experience the potentials and limits of your body. For example, M3, a 28-year-old architect, recorded his experience of one marathon race in his diary:

Marathon is normally considered as a boring and physically-intensive sport in outsiders’ eyes. But after you have participated in it, you would know that it’s a process of communication between you and your body. While your body was extremely tired, it persuaded you to give up. But simultaneously, your brain would generate endorphin that made you excited and joyful. Gradually, you would be addicted to these complex feelings… I think marathon has changed me from within, which manifested in not only the body shape but also the spirit and the energetic state of life. It is a systematic transformation of the self.

M3’s experience is consistent with Shipway and Holloway’s argument [6] that running provides people a source of meaning and a life-changing experience that enable runners to cultivate a confident self. Yet, we further suggest that running as a project of self-exploration is animated by the situated bodily experience in particular “moments.” For example, the bodily experience of “tiredness” and “painfulness” frequently appears in the interviews and dairies. In particular, the negotiation of painfulness during marathon describes the bodily experience of most practitioners. F5, a 34-year-old banker, contended that the painful experience offered her a way to explore the limits of her body, through which she can attain a new understanding of painfulness and the self:

When I reached the last 10 km in my first marathon attempt, I intensively felt that I had pushed my body to its limits. I heavily and slowly moved my legs that went into convulsions. I could clearly hear my breaths and heartbeats. It was definitely painful…But, when I came back from it, I couldn’t help having a second attempt of marathon, to continue experiencing this kind of pain. I realised that pain was just a part of my experience that I didn’t need to avoid. I learned to attain happiness from being in pain during a marathon.

Apart from self-exploration, M14, a 34-year-old manager, explains that the embodied experience of marathon enabled him to reclaim the autonomy of the body and to escape from the patterned lifestyle in the city. For M14, marathon running helped him to rediscover the “true potential” of his body and attain an state of transcendence, in which he detached from the patterned and ordinary self:

After a few years into the job, I had led an increasingly patterned and programmatic lifestyle, nothing had changed. I couldn’t find any passion until I took up marathon running. Running has enabled me to break up this patterned life trajectory that may constrain me in the expected future…Running a marathon felt like riding a roller coaster; it is painful yet exciting while you keep pushing the limits of your body. It seemed masochistic, but If you didn’t do this, you would never know your true potential.

Although marathon is a sport that requires the management and discipline of the body, many correspondents report that self-discipline instead enabled them to reclaim control of their body/life. For example, F9, a 28-year-old company staff member, told us that it helped her attain higher degree of freedom by leading an ordered and self-disciplined lifestyle: “When you engage in marathon, your life becomes more ordered because you pay more attention to managing your body and time and stop staying up late. To maintain a healthy lifestyle, I also had courage to reject many unnecessary parties.” In general, the running body is an important site through which they can transform themselves to attain a certain state of freedom, control, and self-realisation that form the basis for a healthy lifestyle.

### 4.2. Wearable Technology and Digitally-Mediated Body

As Esmonde [31] (p. 809) noted, “the practice of self-tracking can influence a person’s movement through the world while running or walking in important ways.” In particular, self-tracking and wearable technologies can reconfigure the ways runners make sense of the relationship between space and self through a quantitative lens. In this section, we reveal more complex ways of how running body and space are digitally mediated and negotiated. We suggest that running bodies are also shaped by wearable technologies that facilitate, condition, and even structure the ways in which marathon runners manage their own bodies, conduct, and ways of being and extent to which they exercise their agency. According to the interviews and dairies, wearable technologies, such as self-tracking devices, GPS, and running-oriented apps, are widely used among runners. Twenty-five of 29 participants reported that they frequently used wearable devices or other running-oriented apps in running.

On the one hand, for most runners, the use of self-tracking and wearable digital devices is a crucial requirement of scientific running. That is, it is through the digital quantification of the body that runners can scientifically monitor their bodies, avoid risks, and achieve self-betterment. As F1, a 40-year-old manager, noted:

I must use the watch from which I can see the indexes of my body because I think I am a scientific runner. It can help me more efficiently set up my own training plans. I can see the number and intensity of trainings that I have done and I intend to reach. After training, these devices can help you monitor your body—whether your body has re-energised or whether it is ready for the next race.

Wearable technology not only provides an quantitative account of runners’ body but also shapes the ways and rhythms that they interact with the space/place around them while they are running. The word “rhythms” (jiezhou, 节奏) were frequently mentioned by many marathon runners. For them, wearable technology can help them significantly build up the rhythms of running. Edensor and Larsen [5] note that running rhythms is a body’s harmonious relation with the situated environments and the spatial-temporal arrangements in marathon running. These include but are not limited to the control of the speed, breathing, and pulse in accordance with particular phases/environments in marathon [34]. In this sense, wearable technology plays a crucial role in establishing the rhythms. For example, M5, a 23-year-old college student, explained to us the importance of the digitally-mediated rhythms in marathon running:

Sometimes losing your rhythms (jiezhou, 节奏) of running would really affect your mood and lead to frustration…You need to know where you can speed up and where you should preserve your strength. You may face topographies during marathon, so you need to adjust your paces accordingly. The running watch can help you achieve this by offering you in-situ data.

On the other hand, the construction of a quantified body also means that data and digital technologies are not simply “tools” but rather an extension of the body. In other words, digital technologies have homeostatic autonomy that in turn conditions and disciplines human affective capacities [39]. To a certain extent, the digital normalises the disciplined bodies by quantitively guiding runners to overcome or be cautious of the whims (and basically laziness) of the self. As M15, a 28-year-old teacher, suggested, the data becomes an integral part of his body:

I rely on the data stored in my watch. If I forget to wear it, I feel anxious when I am running, because the device can tell you the locations, track your footprints, and record your heartbeat. They are very important for a runner. Without these data, you may be in a dangerous condition that you don’t realise. So, mastering this information is also being responsible to your own body.

Similarly, F4, a 35-year-old manager, highlighted her reliance on the wearable running devices and what counts as “scientific” running:

If you run without the device, you can’t find the problems that may harm you. It’s unscientific. For example, you cannot know whether the strength from your two legs are equal. This may harm your legs if you don’t realise… Without the device, I will feel uncomfortable.

M15 and F4’s running practices indicate that reliance on digital technologies may also lead to an emphasis on data over feelings, confidence, and corporeal sensations. In this sense, wearable technologies can offer a scientific approach to running but also simultaneously structure and limit individuals’ perception and imagination of the embodied potential of the body. In a few situations, digital technologies may decrease the pleasure of running, as data do not always follow runners’ desires [31,32]. As a few correspondents noted, failing to achieve the expectations they set up (e.g., the amount of training) would always upset them. For example, F6 told us that unsatisfied data leads to a feeling of loss and frustration because it influences her confidence and rhythms in marathon running: “When the data shows that you didn’t finish the first half in the expected time, it will definitely thwart your confidence because the second half will be more challenging.” In this sense, the stress imposed by the data also took their focus away from the pleasure in running.

Overall, the embodied and digital practices of marathon running are important “technologies of the self” through which runners constitute a desired and scientific way of health. However, the relationship between body and wearable technology are always mutually constitutive: on the one hand, wearable technology can enhance the potential of the body that help runners to achieve self-betterment and self-exploration; on the other hand, the running body is also shaped and structured by both technological forces that may limit its agency. In what follows, we further elaborate on the collective running body and the formation of embodied atmospheres of health.

### 4.3. Atmospheric Experience of Health

Marathon running is not an isolating sport but rather an atmospheric space in which different bodies, objects, and environments co-produce runners’ situated experience. Many marathon runners highlight that the “atmosphere” (qifen, 气氛) is an important source of their enjoyment that enables them to participate repeatedly. Drawing on the epistemology of actor-network theory, we therefore reveal how the atmospheric space of health is constructed through runners’ interaction with other human bodies (e.g., runners and audiences), non-human objects, and situated environments/landscape (e.g., nature and weather). These atmospheres in turn shape runners’ embodied practices of health. According to our coding, we particularly emphasise the ritualistic, aesthetic, and therapeutic nature of place/nature and how it contributes to generating the “affective atmosphere” of marathons.

The affective atmospheres of marathons are co-produced by, for example, the opening ceremony, the chants and cheers from the audiences, the particular place/landscape that people run across, and the interactions of different runners. These collectively create a sense of ritual that distinguishes marathons from the ordinary. For example, M2, a 30-year-old IT developer, explained how he attains a strong sense of ritual in marathons:

I think the atmosphere of a marathon is something that really puts you in motion. It make you excited immediately. This is quite different from the situations that you ran individually because you can’t feel these atmospheres and especially the sense of ritual—you feel like you are participating in a very especial event.

Similarly, M5, a 22-year-old college student, recorded his accounts of the ritualised atmospheres in a marathon staged in Beijing:

I have been to Beijing three times, but this time is quite different. The starting point of marathon was set up at Tiananmen Square. That really gave me a sense of spectacle and ritual. It made you felt that this particular moments and the spectacular architectures were exclusively designed for you…

As Collins [40] (p. 340) notes, ritualised atmospheres or spaces are generated from the assemblage of the collective bodies in a physical attunement: “When human bodies are together in the same place, there is a physical attunement: currents of feeling, a sense of wariness or interest, a palpable change in the atmosphere.” This is particularly the case in marathons in which different bodies are immerged into a collective affective atmosphere. For example, F9, a 28-year-old company staff, described how this atmosphere serves as an affective force that pushes her body:

When you went to that mood and atmosphere, you would never easily quit even though you were extremely tired. There were quite a lot people around you. Not matter how fast and slow you ran, there were always people that accompanied you. We called each other running fellows regardless of age and gender. We would encourage and take care each other on the road. So, there was an atmosphere there.

The atmospheres of marathons are also formed through runners’ embodied encounter/interaction with particular place, nature, landscape, and environmental conditions. When participants run across/through spaces, they also experience and attach meanings to the situated space/place around them. For example, M4, a 40-year-old teacher, considered marathon as a journey in which he can view the aesthetic landscape in different places across China. Yet, For M4, marathon is not simply a journey because it enables him to interact with the place in a mobile way that he cannot experience in normal tourism:

Marathon is like a journey in that you can view different landscape and experience different cultures in different places of China. But the difference [between marathon and travel] is that you are embracing the landscape while you are running, you are using your foot to measure the land you ran through. For example, I participated a marathon in Yangzhou. That was in March, as the Chinese ancient poetry says: “In the mist and flowers of spring, I journeyed south to Yangzhou” (烟花三月下扬州). When I ran along the West Lake, I can feel the connection with this place. This experience was quite different from that of tourism visitors.

M4’s experiences suggest that marathon running can be seem as the embodied encounters with places. On the one hand, we acknowledge the body’s crucial roles in generating aesthetic experience of place [33]. Yet, on the other hand, the embodied encounter in marathon running is not simply a sensual and visionary “tourist gaze” [41] but rather a mobile practice that can engender new atmospheric and aesthetic perception of space/place.

Many runners also emphasis the role of situated natural environments and especially weather in creating different atmospheric feelings in marathons. As Larsen and Jensen [37] (p. 1) argued, the atmospheres of running is also “mediated by the material sensations of what Ingold [42] terms ‘weather-worlds,’” as “people move in and through the air, sunshine, heat, rain, wind, snow, fog, or icy roads.” For example, M1, a 30-year-old manager, described in his diary how he attained a sense of purification and a therapeutic feeling when he was running in cold and rainy environments:

It was a cold and rainy morning, around nine degrees Celsius. My body hadn’t warmed up even though I had ran away from the starting point for 20 min. When I ran across the city centre, it’s strange that I didn’t see the streets thronged with people and traffics as I expected. At this moment, the city hadn’t yet revived from the night time, peaceful and cool. This somewhat purified me and brought me peacefulness at that moment.

As Schusterman [43] (p. 8) argued, “to focus on feeling one’s body is to foreground it against its environmental background, which must be somehow felt in order to constitute that experienced background.” It is through the situated environments that “an essentially situated, relational, and symbolic self” can be animated and felt. In this case, it is through the ritualistic, atmospheric, and environmental atmospheres from which the marathon, as a journey of self-exploration, is affectively engendered and constituted. The experience and subjectivity of health therefore emerge from these embodied encounters between body and space/place in marathon running.

## 5. Discussion

**Figure 1:**
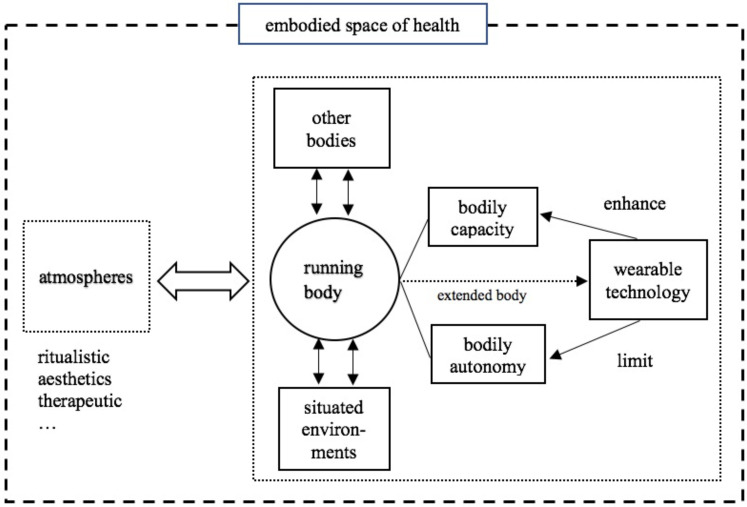
The model of the embodied space of health in marathon running.

In general, this paper suggests that the embodied space of health in marathons emerges through interaction of body, non-human objects (wearable technology), and atmospheres. As we see in Figure 1, the construction of running body provides marathon participants a way to build up the capacity of the body and to reclaim the autonomy of the body. This bodily capacity including not only the biophysical presence of health but also individuals’ project of self-exploration and self-realisation, which are achieved through mobile and disciplined running practice. Running also helps participants to escape from or resist to the patterned and programmatic “social body” so as to reclaim the autonomy of their bodies. Overall, the body can be viewed as a basic spatial unit through individuals act on themselves to attain healthy or desired being of the body.

The qualitative data also show the ways that wearable technology interacts with body and space. Given the intimacy between body and technology, the self-tracking devices can be considered as the extended body of runners. These wearable technologies enhance runners’ bodily capacity by establishing a “quantified self” and by refiguring the ways they make sense of the spaces around them. Yet, wearable technology also in turn constrains runners’ bodily autonomy, as they heavily rely on data and technology to achieve self-betterment. Digital technology, as Esmonde noted, can induce runners to particular assumptions and expectations of their own: “that running should have a purpose beyond pleasure in movement that one can shape their body through data collection and the type of body towards which people aspire, and that improving one’s numbers by running faster and longer is a common-sense goal” [31] (p. 814). This is particularly the case in this paper: that these assumptions instead distract runner away from the pleasure in running.

This paper also offers an account of the diffused and atmospheric spaces of health. We outline the ritualistic, aesthetic, and therapeutic atmospheres that emerge from the interactions between different bodies and between the body and situated environments in marathons. In this sense, we therefore argue that health experience is not simply constructed through biophysical (e.g., the medical definition of health) and discursive processes (e.g., healthism) but also can be captured through the lens of atmospheres. In general, drawing on an actor-network theory and the relational accounts of health, we suggest that the embodied space of health in marathon running is not simply bodily experience per se but rather is the relational space constituted through the interactive effects of body, technology, and atmospheres.

## 6. Conclusions

In this paper, we have shown what is the “embodied space of health” in marathon running and how it is formed through the interactions between body, wearable technology, and space (especially the atmospheres). This paper advances the research of public and environmental health studies by offering a relational and non-representational approach to capturing the spatiality of health experiences. We argue that the effects of health emerges from the bodily, digitally mediated, and atmospheric experiences of running. Different from the research on health and place that tends to associate health with particular qualities of place, the “embodied space of health” highlights the relational and non-representational nature of health that emerges from the relationalities of different bodies and objects. We also note that through particular atmospheres in running, space becomes meaningful places such that the therapeutic effects of places are engendered. Yet, the limitations of this research are also noticeable. Our study cannot fully capture and understand the bodily and atmospheric experiences in running due to the limitation of interviews. Therefore, future studies can use innovative methods, such as mobile methods and qualitative GIS [44], to explore the more complex spatiality of health experience.

## Figures and Tables

**Table 1:** Demographic information of participants.

Number	Gender	Age	Occupation	Number	Gender	Age	Occupation
M1	Male	30	Manager	M16	Male	27	Freelancer
M2	Male	30	IT developer *	M17	Male	41	Doctor
M3	Male	28	Architect	M18	Male	30	Company staff
M4	Male	40	Teacher	M19	Male	50	Constructor
M5	Male	22	Student	M20	Male	28	Entrepreneur
M6	Male	25	Student	F1	Female	40	Manager
M7	Male	40	Teacher	F2	Female	28	Company staff
M8	Male	23	Student	F3	Female	50	Accountant
M9	Male	38	Manager	F4	Female	35	Manager
M10	Male	48	Manager	F5	Female	34	Banker
M11	Male	25	Company staff	F6	Female	26	Banker
M12	Male	24	Student	F7	Female	23	Teacher
M13	Male	29	Company staff	F8	Female	29	Researcher
M14	Male	34	Manager	F9	Female	28	Company staff
M15	Male	28	Teacher				

* Note: IT refers to information technology.

**Table 2:** The data coding of marathon running.

Aggregate Dimensions	2nd-Order Concepts	1st-Order Concepts	Examples of Illustrative Quote
Bodily experience of health	Capacity of the body	Pursuing the healthy and desired body	“I have hyperlipidaemia and fatty liver. So, I decided to change by running marathon.”
		Exploring the potentials and limits of the running body	“That felt like riding a roller coaster, which made you addicted and kept you pushing the limits of your body.”
	Autonomy of the body	Cultivating self-disciplined bodies	“You paid more attention to manage your body and time and stopped staying up late.”
		Resisting social norms	“Running has enabled me to break up this patterned life trajectory.”
Digitally-mediated experience of health	Self-betterment through wearable technology	Establishing quantified self	“I can see the number and intensity of trainings that I have done and I intend to reach.”
		Self-monitoring	“After training, these devices can help you monitor your body.”
	Negotiation of digital agency	Constraints of the wearable technology	“Without the device, I can’t ensure whether I was leading a scientific running. It made me uncomfortable”.
Atmospheric experience of health	Affective atmosphere	Sense of ritual	“You feel validated because of this sense of ritual.”
		Interaction of the bodies	“We would encourage and take care each other on the road.”
		Aesthetic place and landscape	“When you are running, you can experience different beautiful landscapes across China”.
	Therapeutic environments	Nature, urban environment, and weather	“This [environment] somewhat purified me and brought me peacefulness at that comment.”
